# Handgrip strength evaluation in CKD: do we have enough evidence?

**DOI:** 10.1590/2175-8239-JBN-2020-0209

**Published:** 2020-11-11

**Authors:** Viviane O. Leal, Denise Mafra

**Affiliations:** 1 Universidade do Estado do Rio de Janeiro, RJ, Brasil.; 2 Universidade Federal Fluminense, Niterói, RJ, Brasil.

Diagnosis of malnutrition in patients with chronic kidney disease (CKD) is a challenge because there is no "broad and perfect" indicator^1^^,^ 2. In the recently published clinical practice guideline for nutrition in CKD^2^, composite tools, such as the malnutrition-inflammation score (MIS), were highlighted because they include clinical, dietetic, biochemical, and anthropometric variables and, therefore, are more comprehensive.

In addition, the handgrip strength (HGS) was listed as a reliable and straightforward method to evaluate muscle function in these patients, and it can be used as an indirect measure of nutritional status. According to the Guideline: "In adults with CKD 1-5D, HGS may be used as an indicator of protein-energy status and functional status when baseline data (prior measures) are available for comparison"^2^.

HGS measurement is a useful tool for identifying the functional disability and the risk of early mortality^3^ in patients with CKD^4^^,^ 5. However, studies with CKD patients use different cutoff values, making a general conclusion difficult due to a lack of consensus. Thus, a HGS cutoff value should be defined for these patients to guide the clinical practice and for the production of studies that are more conlusive^2^^,^ 4.

More recently, Sostisso and colleagues^6^ have indicated the HGS cutoff point of <14.5kg for women and <23.5kg for men to diagnose malnutrition in Brazilian patients on hemodialysis, having the MIS as a reference. However, the main limitation of this study was the wide age range (18 to 87 years) because it is well known that elderly patients present not only a reduced muscle strength but also an altered motor coordination compared to younger people, which can result in an inaccurate measurement. Therefore, it seems inappropriate that the same HGS cutoff values are applied to young, adult, and elderly patients. However, this study is very important to start the debate on a consensus recommendation of HGS cutoffs for the Brazilian hemodialysis population.

In this context, another study also with Brazilian CKD patients on hemodialysis^7^ reported that mortality was higher in patients with HGS below the sex-age-specific cutoffs (17.8 kg for women <60 years, 13.8 kg for women ⩾60 years, 29.5 kg for men <60 years, and 21.9 kg for men <60 years), showing that it is important to take into account the age of the patient.

**Figure 1:**
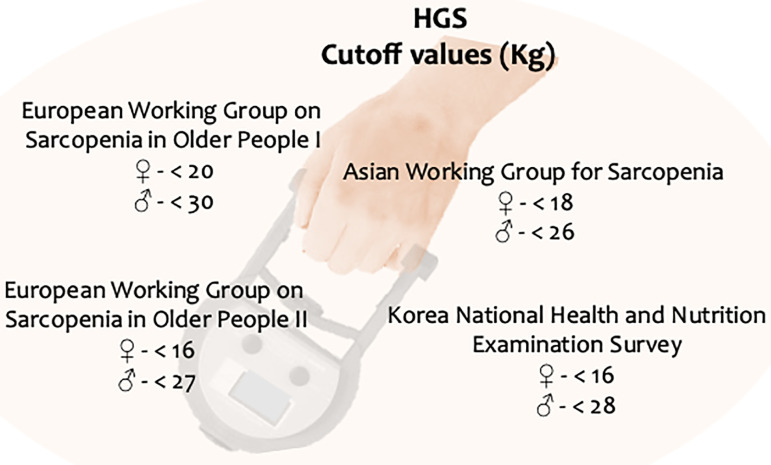
Cutoff values for handgrip strength (HGS) according to different groups. Adapted from Lee & Gong^9^.

In the updated definition of sarcopenia, the European Working Group on Sarcopenia in Older People (EWGSOP2)^8^ proposed a cutoff point of <16kg for women and <27kg for men for probable sarcopenia diagnosis. However, they recommend using regional normative populations for reference (when available) because this guideline was based on European populations, and the HGS varies with stature^8^^,^ 9. Several groups have been working on defining the cutoff for specific populations, as shown in Figure 1.

The definition of cutoff points for HGS can mitigate the anxiety of professionals involved with CKD patients in diagnosing the nutritional status as "normal" or "non-normal". However, the most interesting feature of HGS is its capacity to detect early alterations in nutritional status. Functional tests, such as HGS, are usually the most sensitive and relevant indicators of short-term nutritional status changes and correlate with prognosis and clinical complications^3^^,^ 4 ^,^ 5.

In this sense, the current clinical practice guideline for nutrition in CKD^2^ suggested HGS as a useful indicator of nutritional and functional status when prior measures are available for comparison. That is, follow-up evaluations can be more important than using the measure a single time for classification. The clinical monitoring of patients is more important than their fitting into a cutoff that can be imperfect given the peculiarities of each person, including age and CKD diagnoses, in addition to comorbidities and dialysis vintage.

Additionally, comparisons with cutoffs obtained from different dynamometers types and different test conditions could predispose a confused conclusion/diagnosis, reinforcing the importance of follow-up measures^8^. So far, there is no definition about the best time for measuring CKD patients on hemodialysis (pre- or post-dialysis session or in a non-dialysis day) and there is no standardized technique (choice and position of the arm, the rest time)^2^^,^ 4 ^,^ 9. Moreover, HGS relies on the subjects' motivation, and therefore, professionals must be sufficiently trained in HGS assessment^2^^,^ 4.

In CKD, muscle wasting and sarcopenia have many contributing causes beyond ageing^1^. These causes affect interventions that prevent or delay the development of nutritional abnormalities^8^. In this context, the nutritional evaluation is critical to establish treatment strategies, but HGS evaluation still has several unanswered questions such as: what is the best way to interpret HGS data? The data should be compared to the reference standards or to follow-up measures of the same patient? However, the usefulness of the HGS for assessing the protein-energy nutritional status is undeniable because it is a simple, non-invasive, and reliable method^2^. We believe that it is a common sense that the patient's clinical context should be adapter to the reference standards established, and individual characteristics should be taken into account when using HGS in CKD patients. Moreover, as the new guidelines^2^ recommend, prior measures should be performed for comparisons.
